# The clinical effectiveness of different parenting programmes for children with conduct problems: a systematic review of randomised controlled trials

**DOI:** 10.1186/1753-2000-3-7

**Published:** 2009-03-04

**Authors:** Janine Dretzke, Clare Davenport, Emma Frew, Jane Barlow, Sarah Stewart-Brown, Sue Bayliss, Rod S Taylor, Josie Sandercock, Chris Hyde

**Affiliations:** 1 Unit of Public Health, Epidemiology and Biostatistics, University of Birmingham, Edgbaston, Birmingham, B15 2TT, UK; 2 Unit of Health Economics, University of Birmingham, Edgbaston, Birmingham, B15 2TT, UK; 3 Health Sciences Research Institute, Warwick Medical School, University of Warwick, Coventry, CV4 7AL, UK; 4 PenTAG, Institute for Health Services Research, Peninsula Medical School, Noy Scott House, Royal Devon and Exeter Hospital, Exeter EX2 5DW, UK

## Abstract

**Background:**

Conduct problems are common, disabling and costly. The prognosis for children with conduct problems is poor, with outcomes in adulthood including criminal behaviour, alcoholism, drug abuse, domestic violence, child abuse and a range of psychiatric disorders.

There has been a rapid expansion of group based parent-training programmes for the treatment of children with conduct problems in a number of countries over the past 10 years. Existing reviews of parent training have methodological limitations such as inclusion of non-randomised studies, the absence of investigation for heterogeneity prior to meta-analysis or failure to report confidence intervals.

The objective of the current study was to systematically review randomised controlled trials of parenting programmes for the treatment of children with conduct problems.

**Methods:**

Standard systematic review methods were followed including duplicate inclusion decisions, data extraction and quality assessment. Twenty electronic databases from the fields of medicine, psychology, social science and education were comprehensively searched for RCTs and systematic reviews to February 2006.

Inclusion criteria were: randomised controlled trial; of structured, repeatable parenting programmes; for parents/carers of children up to the age of 18 with a conduct problem; and at least one measure of child behaviour. Meta-analysis and qualitative synthesis were used to summarise included studies.

**Results:**

57 RCTs were included. Studies were small with an average group size of 21. Meta-analyses using both parent (SMD -0.67; 95% CI: -0.91, -0.42) and independent (SMD -0.44; 95% CI: -0.66, -0.23) reports of outcome showed significant differences favouring the intervention group. There was insufficient evidence to determine the relative effectiveness of different approaches to delivering parenting programmes.

**Conclusion:**

Parenting programmes are an effective treatment for children with conduct problems. The relative effectiveness of different parenting programmes requires further research.

## Review

### Introduction

Conduct problems are common and disabling. Based on a survey by the Office of National Statistics (UK) from 1999[1], 5.3% of all children and adolescents between the ages of 5–15 had clinically significant conduct problems, the commonest reason for referral for psychological and psychiatric treatment in childhood [2]. The prognosis for children with conduct problems is poor, with outcomes in adulthood including criminal behaviour, alcoholism, drug abuse, domestic violence, child abuse and a range of psychiatric disorders [3-6].

Conduct problems are costly[7] due to the trauma and psychological problems caused to others who are victims of crime, aggression or bullying, together with the financial costs of services for treatment of both the condition and its long-term sequelae. Services include community youth justice services, prison services, social services, psychiatric, general practice and A&E services, and the costs of unemployment and other benefits. A recent UK study[8] covering a limited selection of these costs suggested that by age 28, costs for individuals with a clinical diagnosis of conduct disorder were 10.0 times higher than for those with no problems (CI: 3.6 to 20.9) and costs for those with conduct problems not meeting diagnostic criteria were 3.5 times higher (CI: 1.7 to 6.2).

### Treatment for conduct problems

Various interventions have been used to treat conduct disorder including behaviour therapy, residential treatment, drugs, family therapy, multisystemic therapy and programmes which aim to improve parenting. The latter are unique in that they are structured, short-term interventions (average of two-hourly weekly sessions over 10–12 weeks) provided in a variety of settings (hospital, community, clinic/office or home) with a group or with individual parents (face-to-face or via telephone). They are directed at parents and reflect an increasing recognition that aspects of parenting such as boundary setting, positive discipline and warm and affectionate relationships are key in the prevention of behaviour problems [9].

A range of professionals can deliver the programmes, including psychologists, therapists/counsellors, social or community workers. In self-administered courses parents are encouraged to view videotapes or read training materials (books and leaflets). In some programmes the index child attends as well as the parents allowing parents to rehearse new skills or therapists to coach parent-child interaction. Some parenting programmes cover additional components such as stress or anger management.

There has been a rapid expansion of group based parent-training programmes over the past 10 years [10] and the provision of parenting programmes is central to the UK governments' social inclusion agenda. A systematic review of existing reviews of the effectiveness of parent training for conduct disorder that were judged to be of high quality using a recognised checklist [11] suggested that parenting programmes are an effective intervention for children with behaviour problems.

Two of these reviews produce summary measures suggesting parent training programmes have a significant positive effect in crime prevention [12] and for non-compliant children [13] although this latter review does not provide any indication of the uncertainty of the effect estimate. One review reports a summary measure suggesting a non significant trend favouring parent training in children 0–3 years [14]. Two reviews do not report summary measures of effectiveness but suggest that parent training has a positive effect on children's behaviour problems, parental well-being and social outcomes [15] and a positive effect for young children with conduct disorder [16].

In addition two recent reviews have investigated moderators of effectiveness of parenting programmes on disruptive child behaviour [17] and on child externalizing behaviour problems [18]. Variables such as socioeconomic status, the inclusion of children in the parenting programme, maternal mental health and individual versus group approaches to delivery moderated effectiveness although these effects tended to be modest.

However these existing reviews have limitations, such as the inclusion of non-randomised studies, the absence of a test for heterogeneity prior to the conduct of a meta-analysis and failure to report confidence intervals. The two reviews investigating moderators of effectiveness both suffer from statistical limitations such as use of small data sets and underestimation of heterogeneity. In addition these existing reviews have largely been restricted to the impact of parenting programmes on specific population sub-groups and have not endeavoured to estimate the overall impact of parenting programmes on children with conduct problems. Further no existing reviews have attempted to compare the relative effectiveness of different types of programmes.

The objective of the current study was therefore to systematically review randomised controlled trials (RCTs) of parenting programmes for the treatment of children (≤ 18 yrs) with conduct problems to investigate i) the overall effectiveness of parenting programmes, and; ii) the relative effectiveness of different approaches to delivery.

## Methods

### Search strategy

Twenty electronic databases (including PsycInfo, MEDLINE, EMBASE and the Cochrane Library) from the fields of medicine, social science and education, and the National Research Register Issue 1 (2006) were searched up to February 2006. There were no language restrictions. In addition citations from previous reviews and included studies were searched and information was requested from manufacturers and experts.

### Inclusion and exclusion

Studies were included if: (a) they were RCTs, (b) the population comprised parents/carers of children up to the age of 18 where at least 50% had a conduct problem (defined using objective clinical criteria, the clinical cut-off point on a well validated behaviour scale or informal diagnostic criteria), (c) the intervention was a structured, repeatable (manualised) parenting programme (any theoretical basis, setting or mode of delivery) and (d) there was at least one standardised outcome measuring child behaviour. Studies where children accompanied their parents to all or some of the sessions were included providing the main focus of treatment was on the parents (i.e. children were present for parental skill rehearsal or assessment). Inclusion of studies was not restricted by child or parental co-morbidity or by type of comparator (e.g. wait list control, different parenting programme or other treatment).

Studies were excluded where the intervention (a) was aimed at prevention rather than treatment; (b) was aimed specifically at children, the whole family as a unit or at teachers; or (c) was non-structured, such as an informal support group or unstructured home visits.

### Quality assessment and data extraction

Potential threats to internal study validity (selection bias, detection bias, performance bias, attrition bias) were assessed using Cochrane Collaboration [19] criteria. Appropriateness of statistical analyses was critically appraised by statisticians. Inclusion and exclusion of studies, data extraction and quality assessment were undertaken in duplicate, with discrepancies being resolved by a third reviewer.

### Data analysis and synthesis

Studies that had used a child-behaviour measure (reported in at least 20% of all studies) and where there was sufficient statistical information were synthesised quantitatively (n = 24 studies). All meta-analyses were undertaken in Stata™ 7.0. Standardised mean differences were derived to take account of the variety of behavioural outcome measures included and random effect models adopted in view of variability of the intervention and target populations across studies. Tests for publication bias (Egger and Begg tests) were also undertaken.

Planned subgroup analyses involved comparisons between different approaches to delivery for four key characteristics: group or individual or self-administered, length of programme (same or different), index child involvement or adjunctive treatment.

In order to look at the evidence from all relevant studies a vote-counting exercise was undertaken to assess the results of included studies that had not used one of the predominant child-behaviour measures or had not provided enough statistical information to be included in the meta-analysis. For the vote-counting exercise a statistically significant (p ≤ 0.05) difference in favour of the intervention was considered a positive outcome, a statistically significant difference in favour of control was considered a negative outcome and no statistically significant difference was considered a neutral outcome. Thirty eight studies reporting 170 child-behaviour outcome measures were included in the vote-counting exercise.

### Ethics approval

Ethics approval was not required.

## Results

**Figure 1:**
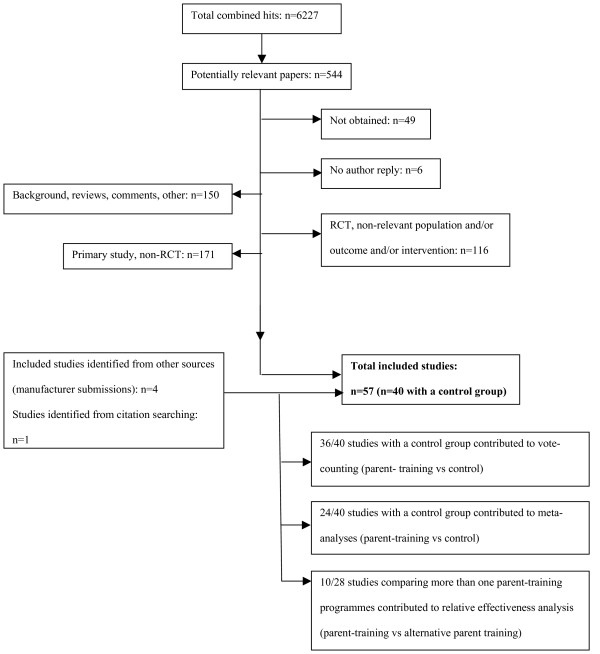
**Inclusion and Exclusion of Studies**.

Figure 1 shows the inclusion and exclusion process. Fifty seven studies were included of which 40 included a control comparison group (no treatment). Twenty eight studies compared parent training with an alternative form of parent training: 17 of these compared parent training with an alternative form of parent training only (no control comparison group) and 11 studies compared parent training with alternative parent training and a control comparison group.

### Intervention characteristics (57 included studies)

The majority of interventions (n = 37) focussed on the parents alone. In 20 studies the intervention(s) involved the child at various levels of intensity, from attendance at all sessions (e.g. Barrett *et al*., 2000[20]), attendance at some sessions for parental skills rehearsal (e.g. 3/8 sessions Pfiffner *et al*., 1990[21]) or observation of children in another setting with feedback to parents during home visits (Sanders & McFarland 2000[22]). Most studies (n = 24) investigated group programmes, of these 23 focussed on parents only. Twenty studies investigated individual based programmes, 15 of which involved index children at some level. The remaining studies investigated self-administered programmes (n = 5) or combinations of group, individual and self-administered programmes (n = 8). Adjunctive treatment such as partner support training, friendship liaison or treatment of depression, was included in the intervention in 8/28 studies comparing two or more parenting programmes. In 3 studies, children were receiving medication for ADHD, all other studies either specifically excluded children receiving concurrent treatment or did not give details of concurrent treatment. No studies comparing parenting programmes with a control group evaluated outcomes past 6 months and only a minority (n = 5) compared 2 alternative interventions between 1 and 3 years.

Concerning 102 parent training programmes and within study variations of these programmes across 57 studies. The majority of programmes (51) were conducted over 10 sessions or less; 17 programmes were 11–20 sessions in length and 10 programmes were greater than 20 sessions in length. For 24 programmes the number of sessions was unclear or not stated. Interventions that were not self-administered (93) were delivered by a variety of professionals: 40 programmes were delivered by psychologists, 1 each delivered by a teacher and a psychiatric nurse and in 51 programmes the professional background of the person delivering the programme was unclear. Social workers were jointly involved in 7 programmes. The great majority of programmes (86) were based on behavioural approaches, 8 on relationship approaches and 4 on both approaches. For 4 programmes the underlying principle was not clear or not stated.

### Population characteristics

Recruitment of populations was via self-referral, media advertisement or fliers in 44 studies; through health professionals or organisations in 10 studies and in 3 studies there was no information on recruitment.

Index children were aged 12 and under or had a mean age < 12 in 49/57 studies and 68% of the agregated study population were male.

Diagnostic criteria (DSM [23]or clinical cut-off on a behavioural scale such as the Eyeberg Child Behaviour Inventory [24]) were used to recruit populations in 48 studies and in 9 studies parent or professional description of child behaviour was used.

In 10 studies some or all children had a diagnosis of Attention Deficit Hyperactivity Disorder (ADHD).

Of 22 studies reporting ethnicity > 70% of study populations were white Caucasian families.

Of the 26 studies reporting family structure more than 30% of index children were in single parent households.

### Quality of research

Few studies reported sufficient information to assess all aspects of quality, and in particular lacked detail about methods of randomisation and allocation concealment. Further detail is provided in [Additional file 1]. No studies were completely bias free, but 4 studies were considered to be of good quality on the basis of only one threat to validity out of a total possible of five [25-28].

No evidence of publication bias was found.

### Effectiveness results

#### Parent-report of outcome

A total of 24 studies contributed a parent-report measure of outcome [25,26,29-50]. Details of these studies can be found in [Additional file 1]. Two instruments were used – Eyberg Child Behaviour Inventory (ECBI): Intensity (n = 20) and the Child Behaviour Checklist (CBCL) (n = 4). The ECBI is a parental report of conduct behavioural problems in children and adolescents that measures the number of difficult behaviour problems (intensity) and the frequency with which they occur [24].

The CBCL is a device by which parents or other individuals who know the child well, rate a child's problem behaviours and competencies [51].

**Figure 2:**
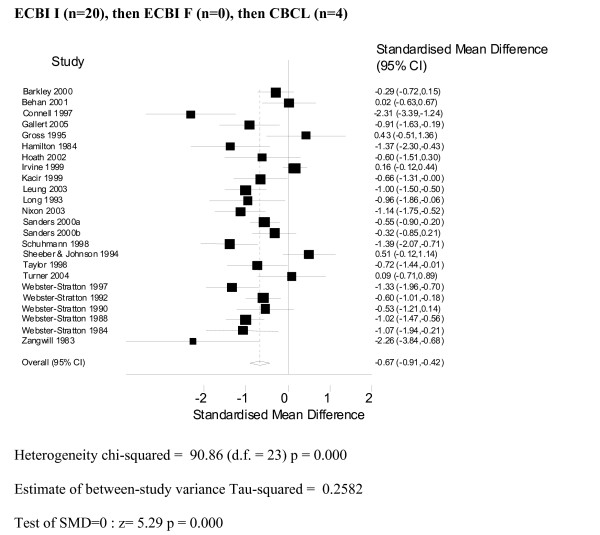
**Meta-analysis ECBI Intensity**.

**Figure 3:**
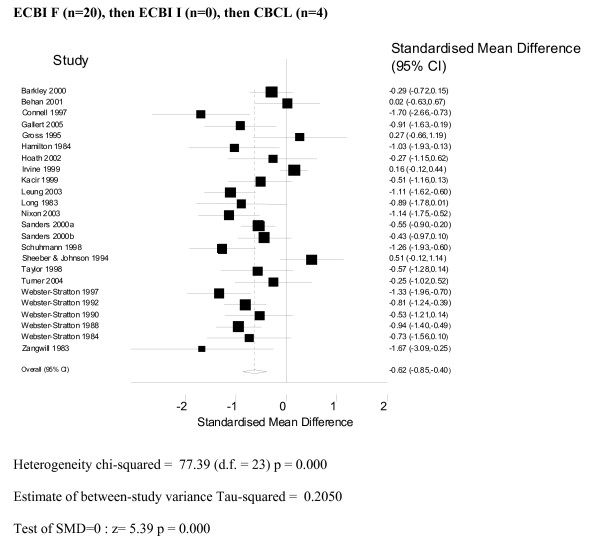
**Meta-analysis ECBI Frequency**.

The results were combined using a random effects model, and the combined results (see Figures 2 and 3) show a significant standardised mean difference favouring the intervention group of -0.67 (95% CI: -0.91, -0.42). The results were similar (SMD -0.62 95% CI: -0.85, -0.40) where the frequency scale (i.e. as opposed to the Intensity scale) of the Eyberg Child Behaviour Inventory was used as the main outcome.

#### Independent assessment of outcome

**Figure 4:**
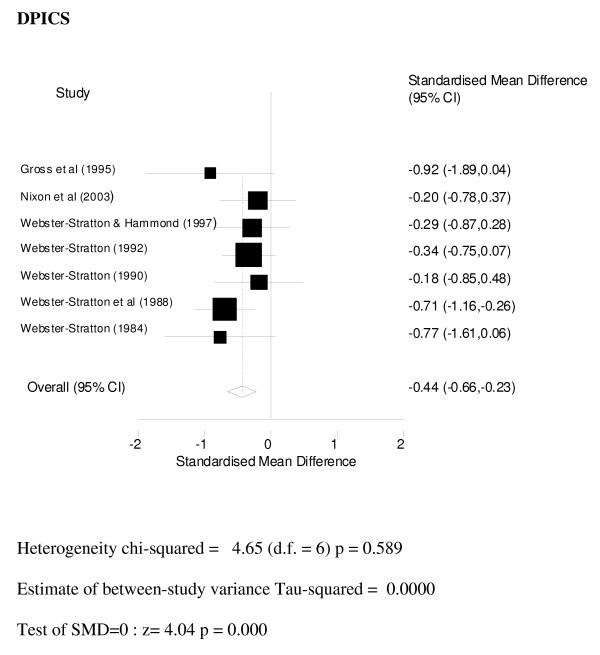
**Meta-analysis DPICS**.

Only 7 studies provided independent assessments of outcomes all of which were undertaken using the Dyadic Parent Interactive Child Scale (DPICS). DPICS is designed for use in assessing the quality of parent-child social interaction. Interaction between parent and child in three standard situations that vary in the degree to which parental control is required is observed and coded by an independent observer behind a two-way mirror [52]. DPICS scores were combined using a random effects model and the combined data (see Figure 4) show a significant standardised mean difference favouring the intervention group of SMD -0.44 (95% CI: -0.66, -0.23).

#### Vote Counting

The results of the vote-counting supported the results of the meta-analysis.

Of 170 child behaviour outcomes measured across 36 studies, 59% were statistically significant and favoured parenting programme over control, with the remaining outcomes showing no statistically significant difference (a neutral outcome). No study demonstrated a less favourable outcome for parent-training compared to control.

#### Relative effectiveness of different approaches to delivery

28 included RCTs compared one parenting programme with another. Most studies were small and none of the studies reported a power calculation to estimate the number of individuals required in order to detect a significant difference in effect for the outcomes measured. Only 10 studies directly compared programmes that differed in only one of the four key characteristics: delivery approach (group, individual or self-administered), length of programme, child involvement and adjunctive treatment (or none)[21,22,39,47,48,53-57]. Comparisons possible were: 3 studies with treatment arms differing only in the approach (group, individual or self-administered), 2 studies differing only in number of sessions and 5 studies differing only in adjunctive treatment. Of 26 behavioural measure comparisons used across these 10 studies only 4 were reported as significantly different. These are detailed in [Additional file 2].

## Discussion

These results show that using both parent-report and independent observations of outcome, parenting programmes are effective in improving conduct problems. Independent observations of change were on the whole smaller than parent-report (SMD of 0.4 compared with 0.7), and very few (7/25) of the included studies had provided an independent assessment of outcome.

There was insufficient evidence to show clear superiority of any one approach to delivery. Many of the comparisons that were undertaken were invalidated by the fact that more than one of the four key characteristics (i.e. group versus one to one, length; child involvement; adjunctive treatment) was varied. Of the ten studies that compared programmes, which varied in only one of the key characteristics, few differences were identified. This is most likely to be due to inadequate power in this analysis.

There may be some restrictions in terms of the generalisability of these findings, due to the involvement in many studies of parents who had self-referred. Similarly, due to the case-mix in many trials there is also some uncertainty regarding the families that would most benefit from this form of treatment.

Our review was restricted to a limited number of behavioural outcomes and we were unable to exploit the full range of behavioural outcome measures used across included studies and for some studies reporting of multiple measures of child behaviour in the meta-analysis. Other reviews have suggested that parenting programmes can have a significant impact on parent psychosocial well-being including stress and self-esteem[58], and that there may be some benefit of such programmes irrespective of ethnic group[59].

Further RCTs comparing different approaches are still needed, focusing in particular on those features that are likely to influence cost as well as effect, such as group versus individual programmes. There is also a need to compare the effectiveness of different programmes in primary studies.

Uncertainty remains regarding the importance of the improvements in child behaviour scores and how these improvements translate into clinically meaningful outcomes. Those who remain sceptical that the demonstrated changes in conduct problems translate into important gains in health and quality of life will point to the need for research quantifying the relationship between change in child behaviour scores and health utility in the index child as well as parents, siblings and peers. Research addressing the long-term impact of parenting programmes is also required.

Work on cost-effectiveness carried out as part of the previous HTA report on this topic[60] and by the Decision Support Unit at the National Institute for Health & Clinical Excellence (NICE) [61] suggests that group-clinic based parenting programmes are likely to be cost-effective or may lead to cost-savings through avoidance of alternative treatment.

### Limitations of the review

While we conducted the review using established criteria [62] it is impossible to exclude certain sources of bias, particularly the possibility of having overlooked eligible studies. Furthermore, as a result of the data available it was not possible to incorporate the findings from all of the studies into the meta-analyses. As noted above, there was also a lack of independent assessments of the presence and size of improvements in conduct problems. Our application of strict inclusion criteria with respect to the structured and repeatable nature of the parenting programme interventions included in this review aimed to ensure that included interventions were similar enough in nature to be pooled in a meta-analysis. In addition the sub-group analysis did not demonstrate any measurable difference in effectiveness according to some aspects of intervention delivery. Nevertheless we cannot rule out the possibility that variation in effectiveness of individual programmes has not been detected.

## Conclusion

We conclude that on balance, parenting programmes are an effective treatment for children with conduct problems. The relative effectiveness of different parenting programmes requires further research.

### Summary points

• Conduct problems among children and adolescents are associated with high psychological and financial costs and with poor prognosis if left untreated

• Parenting programmes are short-term, structured interventions, which have in previous reviews been shown to be effective in treating conduct problems in certain groups of children

• Our systematic review identified 57 randomised controlled trials, which compared parenting programmes to a wait list control or to an alternative form of parenting programme or other treatment

• There was a consistent trend across all studies showing a benefit from parenting programmes; meta-analysis of the most commonly reported child behaviour outcomes showing statistically significant improvements

• There was insufficient evidence to directly determine the relative effectiveness of one type of parenting programme delivery approach over another

• Parenting programmes are an effective treatment for children with conduct problems

## Abbreviations

RCT: randomised controlled trial; SMD: standardised mean difference.

## Competing interests

The authors declare that they have no competing interests.

## Authors' contributions

All authors contributed to protocol development. SB contributed to the development and running of search strategies. JD, CD, EF, CH, JB contributed to inclusion and exclusion of studies. JD, CD, EF, JB, RT, JS contributed to data extraction. JD, CD, RT, CH, JS, JB, SS-B contributed to clinical effectiveness analysis. JD, CD, CH, JB, EF, SS-B contributed to interpretation of effectiveness data and discussion. RT, JS gave statistical advice. JB, SS-B gave clinical advice. CH is the guarantor.

## Supplementary Material

Additional file 1 Characteristics of 24 RCTs included in the meta-analysis. The table provides information about study population characteristics; details of intervention and control groups; main results; quality assessment of studies and the outcome measure contributing to the meta-analysis. Click here for file

Additional file 2 Relative effectiveness of parenting programmes. The table provides information about 10 studies directly comparing parenting programmes differing in only one of 4 key characteristics (delivery approach; programme length; child involvement and adjunctive treatment). Information includes type of comparison; child behaviour outcome measures demonstrating a significant difference between comparison groups; numbers of children in each comparison group. Click here for file
