# Supplementary material for: The clinical effectiveness of different parenting programmes for children with conduct problems: a systematic review of randomised controlled trials
Source: Child Adolesc Psychiatry Ment Health. 2009 Mar 4;3:7. doi: 10.1186/1753-2000-3-7 (PMC2660289; doi:10.1186/1753-2000-3-7)
Supplement: Additional file 1 — Characteristics of 24 RCTs included in the meta-analysis. The table provides information about study population characteristics; details of intervention and control groups; main results; quality assessment of studies and the outcome measure contributing to the meta-analysis. [file 1753-2000-3-7-S1.doc]

# Table 1 Characteristics of 24 RCTs included in the meta-analyses

| **Study** | **Population^a^** Diagnostic criteria (child) N=number of index children | **Intervention/Control**  Group, individual or self-administered | **Main results**  ↔=no statistically significant difference between treatment and control  ↑= statistically significant difference in favour of treatment | **Quality^b^**  Number of threats to validity | **Outcome measures used in meta-analyses^c^** |
| --- | --- | --- | --- | --- | --- |
| Barkley et al., 2000, USA [25] | DSM criteria for ODD or scale  N=158 | • Parenting programme -group  • No treatment control | ↔ 8/8 outcomes | 1 (S) | CBCL |
| Behan et al., 2004, Ireland [29] | DSM criteria for CD or ODD or descriptive  N=50 | • Parenting programme -group  • Wait list control | ↔ 3/3 outcomes | 3  (S, P, AT) | CBCL |
| Connell et al., 1997, Australia [26] | DSM criteria for ODD or CD and/or scale  N=23 | • Parenting programme –individual (parent initiated telephone calls)  • Wait list control | ↑ 2/3 outcomes  ↔1/3 outcomes | 1 (S) | ECBI-F; ECBI-I |

Table 1 contin.

| Gallart & Matthey, 2005, Australia [30] | Scale  N=54 | • Parenting programme - group (Triple P)  • Parenting programme - group (modified group Triple P)  • Wait list control | ↔1/1 outcome (Triple P versus control)  ↑ 1/1 outcome (modified group Triple P) | 3 (S, P, AT) | ECBI-I |
| --- | --- | --- | --- | --- | --- |
| Gross et al., 1995, USA [31] | Scale  N=24 | • Parenting programme -group  • Wait list control | ↑ 1/3 outcomes  ↔2/3 outcomes | 3 (S, P, AT) | DPICS; ECBI-F; ECBI-I |
| Hamilton & MacQuiddy, 1984, USA [32] | Scale  N=27 | • Parenting programme -self-administered (with Signal Seat)  • Parenting programme -self-administered (seat without signal attachment)  • Wait list control | Signal seat versus control:  ↑ 3/4 outcomes  ↔1/4 outcomes  Seat with no attachment versus control:  ↑ 1/4 outcomes  ↔3/4 outcomes | 3 (S, P, AT) | ECBI-F; ECBI-I |

Table 1 contin.

| Hoath & Sanders, 2002, Australia [33] | Scale and all ADHD  N=21 | • Parenting programme -group  • Wait list control | ↑ 1/4 outcomes  ↔3/4 outcomes | 2 (S, AT) | ECBI-F; ECBI-I |
| --- | --- | --- | --- | --- | --- |
| Irvine et al., 1999, USA [34] | Scale  N=303 | • Parenting programme -group  • Wait list control  3 | ↑ 2/2 outcomes | 3 (S, P, AT) | CBCL |
| Kacir & Gordon, 1999, USA [35] | Scale  N=38 | • Parenting programme -self-administered  • Wait list control | ↑ 2/2 outcomes | 3 (S, P, AT) | ECBI-F; ECBI-I |
| Leung et al., 2003, Hong Kong [36] | Descriptive  N=91 | • Parenting programme –group (Triple P)  • Wait list control | ↑ 4/4 outcomes | 3 (S, P, AT) | ECBI-F; ECBI-I |

Table 1 contin.

| Long et al., 1993, USA [37] | Scale and all ADHD  N=32 | • Parenting programme -self-administered  • Standard treatment | ↑ 3/5 outcomes  ↔2/5 outcomes | 3.5 (S, P, AT, AN) | ECBI-F; ECBI-I |
| --- | --- | --- | --- | --- | --- |
| Nixon et al., 2003, Australia [38] | DSM criteria for ODD and scale  N=67 | • Parenting programme -individual (standard)  • Parenting programme – individual (abbreviated)  • Waitlist control | ↑ 6/12 outcomes  ↔ 6/12 outcomes | 3 (S, P, AT) | DPICS; ECBI-I |
| Sanders et al., 2000 (a), Australia [39] | Scale  N=305 | • Parenting programme -self-administered  • Wait list control | ↑ 2/3 outcomes  ↔1/3 outcomes | 2 (S, P) | ECBI-I |

Table 1 contin.

| Sanders et al., 2000 (b), Australia [40] | Scale  N=56 | • Parenting programme -self-administered  • Wait list control | ↑ 1/2 outcomes  ↔1/2 outcomes | 3 (S, P, AT) | ECBI-F; ECBI-I |
| --- | --- | --- | --- | --- | --- |
| Schuhmann et al., 1998, USA [41] | DSM criteria for ODD  N=64 | • Parenting programme – individual (Parent Child Interaction Training)  • Wait list control | ↑ 5/6 outcomes  ↔1/6 outcomes | 3 (S, P, AT) | ECBI-F; ECBI-I |
| Sheeber & Johnson, 1994, USA [42] | Scale  N=40 | • Parenting programme -group  • Wait list control | ↑ 2/2 outcomes | 3 (S, P, AT) | CBCL |
| Taylor et al., 1998, USA [43] | Scale  N=110 | • Parenting programme-group  • Control | ↑ 2/6 outcomes  ↔4/6 outcomes | 3.5 (S, P, AT, AN) | CBCL; ECBI-F; ECBI-I |

Table 1 contin.

| Turner and Sanders, 2004, Australia [44] | Scale  N=30 | • Parenting programme -individual  • Wait list control | ↑ 2/6 outcomes  ↔4/6 outcomes | 3 (S, P, AT) | ECBI-F; ECBI-I |
| --- | --- | --- | --- | --- | --- |
| Webster-Stratton & Hammond, 1997, USA [45] | DSM criteria for ODD and/or CD  N=97 | • Parenting programme -group  • Wait list control | ↑ 4/6 outcomes  ↔2/6 outcomes | 2 (P, S) | CBCL; DPICS; ECBI-I |
| Webster-Stratton, 1992, USA [46] | Scale  N=100 | • Parenting programme -self-administered (in group setting)  • Wait list control | ↑ 4/6 outcomes  ↔2/6 outcomes | 3 (S, P, AT) | CBCL; DPICS; ECBI-F; ECBI-I |
| Webster-Stratton, 1990, USA [47] | Scale  N=47 | • Parenting programme -self-administered (in group setting)  • Parenting programme -self-administered (in group setting, with additional therapist contact)  • Wait list control | Group setting versus control:  ↑ 1/4 outcomes  ↔ 3/4 outcomes  Group plus therapist versus control:  ↑ 1/4 outcomes  ↔ 3/4 outcomes | 3 (S, P, AT) | CBCL; DPICS; ECBI-I |

Table 1 contin.

| Webster-Stratton et al., 1988, USA [48] | Scale  N=114 | • Parenting programme -self-administered (videotape training in group setting)  • Parenting programme -group (videotape training plus group discussion)  • Parenting programme -group (discussion)  • Wait list control | Videotape in group versus control:  ↑ 4/6 outcomes  ↔ 2/6 outcomes  Videotape plus discussion versus control:  ↑ 6/6 outcomes  Discussion versus control:  ↑ 6/6 outcomes | 3 (S, P, AT) | CBCL; DPICS; ECBI-F;  ECBI-I |
| --- | --- | --- | --- | --- | --- |
| Webster-Stratton, 1984, USA [49] | Descriptive  N=40 | • Parenting programme -group  • Wait list control | ↑ 4/5 outcomes  ↔ 1/5 outcomes | 3 (S, P, AT) | CBCL; DPICS; ECBI-F;  ECBI-I |
| Zangwill, 1983, USA [50] | Scale  N=15 | • Parenting programme - individual  • Waitlist control | ↑ 4/7 outcomes  ↔ 3/7 outcomes | 3 (S, D, AT) | ECBI-F; ECBI-I |

**Notes to table 1**

^a^ DSM = formal diagnosis using Diagnostic and Statistical Manual of Mental Disorders; CD=conduct disorder; ODD=oppositional defiant disorder; ADHD=attention deficit hyperactivity disorder; scale=child above (clinical) cut-off level on a child behavioural scale (such as the Eyberg Child Behaviour Inventory); descriptive=no formal diagnostic criteria used

^b^ Number of threats to validity (maximum 5) in following areas: selection bias (S), performance bias (P), detection bias (D), attrition bias (AT) and analysis (AN); fewer threats to validity indicate better study quality and/or quality of reporting

^c^ ECBI-F,I=Eyberg Child Behaviour Inventory-Frequency, Intensity; CBCL=Child Behaviour Checklist; DPICS= Dyadic Parent-Child Interaction Coding System
