# Supplementary material for: The clinical effectiveness of different parenting programmes for children with conduct problems: a systematic review of randomised controlled trials
Source: Child Adolesc Psychiatry Ment Health. 2009 Mar 4;3:7. doi: 10.1186/1753-2000-3-7 (PMC2660289; doi:10.1186/1753-2000-3-7)
Supplement: Additional file 2 — Relative effectiveness of parenting programmes. The table provides information about 10 studies directly comparing parenting programmes differing in only one of 4 key characteristics (delivery approach; programme length; child involvement and adjunctive treatment). Information includes type of comparison; child behaviour outcome measures demonstrating a significant difference between comparison groups; numbers of children in each comparison group. [file 1753-2000-3-7-S2.doc]

Table 2: Relative effectiveness of parenting programmes

| **Study** | **Comparison** | **Child behaviour outcomes showing a significant difference (out of all child behaviour outcomes measured)** | **Patient numbers in parenting programme 1** | **Patient numbers in parenting programme 2** |
| --- | --- | --- | --- | --- |
| Study arms differ in setting only: group, individual or self-administered (same number of sessions, no difference in child involvement or adjunctive treatment) | | | | |
| Raue & Spence, 1985 [55] | Group versus individual | 0/2 | Group n=9 | Individual n=9 |
| Webster-Stratton, 1990 [47] | Self-administered versus combination of self-administered/ individual | 1/4  ECBI-no difference  CBCL-no difference  PDR-no difference  DPICS-significantly less deviant behaviour with self-administered/individual | Self-administered n=17 | Self-administered/ individual n=14 |

Table 2 contin: Relative effectiveness of parenting programmes

| Webster-Stratton et al., 1988 [48] | Self-administered versus group versus combination of self-administered/group | 1/6  ECBI intensity- significantly less frequent behaviour problems with self-administered/group compared to self-administered only  ECBI problem-no difference  CBCL-no differences  PDR-no difference  DPICS-no difference  PBQ-no difference | Self-administered n=27 | Self-administered/ group n=27  Group n=24 |
| --- | --- | --- | --- | --- |
| Study arms differ in number of sessions only (same setting, no difference in child involvement or adjunctive treatment) | | | | |
| Sanders et al., 2000 [39] | Difference in number of sessions | 0/3 | 10 hours of training n=76 | 14 hours of training n=75 |
| Sanders & Christensen, 1985 [54] | Difference in number of sessions | Differences between groups not stated | 9 hours of training n=10 | 12 hours of training n=10 |

**Table 2 contin: Relative effectiveness of parenting programmes**

| Study arms differ in adjunctive treatment only (same setting, no difference in child involvement or number of sessions) | | | | |
| --- | --- | --- | --- | --- |
| Dadds & McHugh, 1992 [55] | Adjunctive treatment: ally support training | 0/3 | Parenting programme  n= assume 11 (22 total) | Parenting programme plus ally support training n=assume 11 (22 total) |
| Dadds et al., 1987 [56] | Adjunctive treatment: partner support training | 0/2 | Parenting programme n=12 | Parenting programme plus partner support training n=12 |
| Sanders & McFarland 2000 [22] | Adjunctive treatment: cognitive therapy for depression | 0/2 | Parenting programme n=24 | Parenting programme plus cognitive therapy for depression n=23 |
| Wahler et al., 1993 [57] | Adjunctive treatment: synthesis teaching (with or without friendship liaison) | 1/2  SOC-R, clinic setting-no results for randomised groups  SOC-R, home setting- significantly less aversive child behaviour in the group with added synthesis teaching at second follow-up (12 months) | Parenting programme n=10 | Parenting programme plus synthesis teaching n=19 |

Table 2 contin: Relative effectiveness of parenting programmes

| Pfiffner et al., 1990 [21] | Adjunctive treatment: social problem solving skills | 1/2  CBCL- no significant difference between groups post-treatment; significantly fewer child behaviour problems in the problem solving group at 4 months  Observation of deviant child behaviour-no difference | Parenting programme n=7 | Parenting programme plus social problem solving skills n=6 |
| --- | --- | --- | --- | --- |
| Study arms differ only in child involvement (same setting, no difference in adjunctive treatment or number of sessions) | | | | |
| No studies identified | Child involvement | N/A |  |  |

SOC-R=Standardised Observation Codes-Revised

PDR=Parent Daily Report

PBQ= Behar Preschool Behaviour Questionnaire

CBCL=Child Behaviour Checklist

ECBI (I, F)= Eyberg Child Behaviour Inventory (Intensity, Frequency)

DPICS= Dyadic Parent-Child Interaction Coding System
